# Place of death and health care utilization for people in the last 6 months of life in Switzerland: a retrospective analysis using administrative data

**DOI:** 10.1186/1472-6963-13-116

**Published:** 2013-03-25

**Authors:** Oliver Reich, Andri Signorell, André Busato

**Affiliations:** 1 Department of Health Sciences, Helsana Group, Zürichstrasse 130, Dübendorf CH-8600, Switzerland; 2 Institute of Public Health, Medical Decision Making and HTA, Department of Public Health and Health Technology Assessment, UMIT - University for Health Sciences, Medical Informatics and Technology, Eduard Wallnöfer Zentrum 1, Hall in Tyrol A-6060, Austria; 3 Institute of Social and Preventive Medicine, Health Services Research, University of Bern, Finkenhubelweg 11, Bern, CH-3012, Switzerland; 4 Institute of General Practice and Health Services Research, University of Zurich, Pestalozzistrasse 24, Zurich, CH-8091, Switzerland

**Keywords:** Switzerland, End-of-life, Health care utilization, Place of death

## Abstract

**Background:**

There is a growing interest in examining the current state of care and identifying opportunities for improving care and reducing costs at the end of life. The aim of this study is to examine patterns of health care use at the end of life and place of death and to describe the basic characteristics of the decedents in the last six months of their life.

**Methods:**

The empirical analysis is based on data from 58,732 Swiss residents who died between 2007 and 2011. All decedents had mandatory health insurance with Helsana Group, the largest health insurer in Switzerland. Descriptive statistical techniques were used to provide a general profile of the study population and determinants of the outcome for place of death were analyzed with an econometric approach.

**Results:**

There were substantial and significant differences in health care utilization in the last six months of life between places of death. The mean numbers of consultations with a general practitioner or a specialist physician as well as the number of different medications and the number of hospital days was consistently highest for the decedents who died in a hospital. We found death occurred in Switzerland most frequently in hospitals (38.4% of all cases) followed by nursing homes (35.1%) and dying at home (26.6%). The econometric analysis indicated that the place of death is significantly associated with age, sex, region and multiple chronic conditions.

**Conclusions:**

The importance of nursing homes and patients’ own homes as place of death will continue to grow in the future. Knowing the determinants of place of death and patterns of health care utilization of decedents can help decision makers on the allocation of these needed health care services in Switzerland.

## Background

Medical care in the final months of life account for a considerable share of health care expenditures (HCE) in comparison to other years [1-6]. Therefore, issues around end-of-life health care have been gaining increasing attention among both policy-makers and researchers. Concerns have been raised over the substantial costs at the end of life and high costs are often interpreted as a result of unnecessary medical procedures trying to keep people alive, irrespective of the preferences of patients and their relatives. Previous research shows that a higher volume of care in terms of higher spending and high-intensity treatment in the last year of life does not produce better outcomes for patients [7-12]. Various studies on end-of-life care also focused on the aspect of place of death and the factors associated with the site of death [13-18]. Other studies have typically concentrated on the relationship between age and health care expenditure [19-24], partly revealing the importance of time-to-death as an important determinant of future HCE [25-29]. Besides examining the location of death and health expenditures, regional variations as well as health care settings at the end of life have also been used to reflect inappropriate care settings as individuals are approaching death. Studies of health care utilization in end-of-life care differ between regional areas and have yielded interesting results [30-34] such as differing utilization pattern depending on available health service resources in rural versus urban areas.

However, little information exists on how constantly growing health care expenditures and health care utilization are distributed across various places of death for patients in Switzerland. Hence, there is a growing interest in examining the current state of care in various settings and there is a need to identify opportunities for improvement and reducing costs at the end of life without compromising the quality of care. To our knowledge, only a few studies have investigated the situation for end-of-life patients in Switzerland. Colombier et al. [35] investigated the impact of population ageing on HCE and concluded that proximity to death is of marginal importance. Moreover, morbidity outweighs mortality as a factor of higher HCE. Official data on the place of death have not been updated by the Swiss Federal Statistical Office (SFSO) since 1987. Therefore, Fischer et al. [36] estimated predictors for the place of death in Switzerland for the year 2001. Hospital deaths occurred most frequently followed by homes for the elderly and dying at home, implying that the relevance of homes for the elderly as a site of death will increase in the future. Two other studies examined the differences in end-of-life decision-making [37,38] in Switzerland and the results suggest that decision-making is related to cultural factors and to the care setting where people die.

This study contributes to the debate on the future development of end-of-life care using administrative health data. The study aims to examine patterns of health care use and place of death at the end of life and explores the basic characteristics of the decedents in their last six months of life.

## Methods

### Data source and sample

Our retrospective analysis is based on administrative data from the health care insurance group Helsana, the largest health insurer in Switzerland, which provides 1.3 million individuals with mandatory health insurance. Individual information on the date of death was used as the inclusion criteria for the analysis and the study population comprised 58,732 Swiss residents who died between 2007 and 2011. It is reasonable to assume that this sample is highly reliable as administrative claims data collected by insurers cover nearly all health care invoices. Deaths due to accidents and suicides are not included in our sample. Unfortunately this information as well as clinical data (e.g. diagnosis or cause of death) is not available in the Swiss health insurer database. We examined health care use and cost for the entire cohort of decedents for the six months immediately prior to death in one of the years 2007 to 2011. A major aspect was to examine where the insured person died; we therefore classified decedents into three mutually exclusive categories: (1) those who died in a hospital, (2) those who died in a nursing home and (3) those who died at home. Geographic classification of place of death was drawn from yearly reports published by the Swiss Federal Statistical Office (SFSO).

### Descriptive analysis

Descriptive statistical techniques were used to provide a general profile of the study population. These data were presented as means and standard deviations (sd) in the case of continuous variables and as percentages in case of categorical variables. We excluded missing values from our descriptive analyses and reported the number of available records. We examined patterns of health care use per decedent in the last six months of life for the following indicators: physician visits, hospital days, nursing home days, number of prescription drugs and home care costs (this was used as a proxy for home care visits given that data were available).

Furthermore, differences between the three groups with respect to place of death in terms of demographics, insurance coverage, morbidity and health care utilization were analyzed with a nonparametric analysis of variance (Kruskal-Wallis test for continuous variables and chi-square tests for categorical variables). Cramer’s V was applied in order to measure the association of the variables.

### Modeling procedures

We developed several statistical models to evaluate the major outcome of care during the last six months of life: place of death. Place of death was defined according to the aforementioned three categories.

In order to assess patient-level effects the following independent variables were included in the models: age, sex, supplementary private hospital insurance coverage, place of residence (city, agglomeration, rural^a^), insurance contract under a managed care model, deductible class, and number of chronic medical conditions identified using pharmaceutical cost groups (PCG). Deductibles are obligatory for all Swiss residents and range from 300 to 2,500 Swiss francs per year. The standard deductible is 300 Swiss francs, but insured persons can choose a higher deductible (500, 1500, 2000, 2500 Swiss francs) in exchange for reduced premiums. PCGs are frequently used as an individual marker for a specific chronic condition [39]. Our classification of PCGs is based on Beck [40] and distinguishes between 13 different groups^b^. We summarized all PCGs for each individual and coded an independent dummy variable as 1 for three or more chronic conditions and 0 for less than three chronic conditions. Furthermore, the intensity of medical treatment for each individual patient was captured. These variables describe health care utilization in the last six months of life per decedent: number of consultations with a general practitioner, number of consultations with a specialist physician, number of days in hospital, length of stay in days in nursing home, number of different ATC (Anatomical Therapeutic Chemical) codes^c^ prescribed (outpatient only) and home care costs. To take into account differences between Latin i.e. French- or Italian-speaking cantons (Fribourg, Geneva, Jura, Neuchatel, Ticino, Vaud and Valais) and German-speaking cantons, we included a dummy variable (1 if Latin canton, 0 if otherwise).

Associations between place of death and patient-level variables were assessed by means of a multinomial logistic regression. We used a log transformation of the independent variables^d^ to estimate the effects. Equation (1) depicts the model used:

(1) $$\begin{array}{l} \text{log}\langle \frac{P\langle Y_{i}=k\rangle }{P\langle Y_{i}=0\rangle }\rangle =\beta_{0}+\beta_{1}\;\mathrm{AG}E_{i}+\beta_{2}\;\mathrm{SE}X_{i}\\ \;+\beta_{3}\;\text{PRI}V_{i}+\beta_{4}\;MC_{i}\\ \;+\beta_{5}\;\mathrm{DE}D_{i}+\beta_{6}\;\mathrm{RE}G_{i}\\ \;+\beta_{7}\;\text{MMOR}B_{i}+\beta_{8}\;\mathrm{AT}C_{i}\\ \;+\beta_{9}\;\text{log}{\left(\mathrm{GP}+1\right)}_{i}\\ \;+\beta_{10}\;\text{log}{\left(\text{SPEC}+1\right)}_{i}\\ \;+\beta_{11}\;\text{log}{\left(\text{HOMC}+1\right)}_{i} \end{array}$$

Where:

Y: Place of death, where dying at home is the according reference value

AGE: Age at time of death in years

SEX: Sex of patient: dummy variable equal to 1 if decedent was female and 0 if male

PRIV: Supplementary private hospital insurance: dummy variable equals 1 if decedent possessed additional private hospital insurance coverage and 0 in all other cases

MC: Managed care health plan type: dummy variable equals 1 if member chose a managed care health plan and 0 in all other cases

DED: Deductible class: dummy variable equals 1 if insured person chose a deductible higher than Swiss francs (CHF) 500 and 0 in all other cases

REG: Region: we defined two dummy variables according to the domicile of the insured person, where the city is the according reference value: Agglomeration area of residence = AGGLO: dummy variable equals 1 if insured person lives in the agglomeration and 0 in all other cases; Rural area of residence = RURAL: dummy variable equals 1 if insured person lives in a rural area and 0 in all other cases

MMORB: Multiple chronic conditions: dummy variable equals 1 if insured person showed more than two chronic conditions and 0 in all other cases

ATC: Number of different medications

GP: Number of consultations with a general practitioner GP

SPEC: Number of consultations with a specialist physician

HOMC: Costs of home care

The strength of associations was measured by the odds ratio (OR) and the respective 95% confidence intervals (CI). Interaction terms of importance were assessed and when significant, a stratified analysis was performed. We estimated a model with patient-level predictors in order to quantify the relative contributions of patient-level characteristics to the place of death. The proportion of variance was defined as McFadden adjusted R-square for the logistic regression; this is helpful in the model building stage as a statistic to evaluate competing models. An assessment of the total model quality was obtained by taking the highest amount of explained variance of the outcome variable.

## Results

### Population characteristics

Table 1 presents sample characteristics of the 58,732 decedents included in our analysis. Women accounted for 53.6% of the total study population. The mean age at time of death was 79.3 years (median 83.0). For women, the mean age of death was 82.2 years (median 82.2), for men 76.0 years (median 76.0). There were differences between the shape of the distribution of age at time of death between the sexes. Men show a larger variability (standard deviation 14.9, interquartile range (IQR) 17) compared to women (sd 13.1, IQR 14).

**Table 1:** Descriptive patient characteristics included in analysis, 2007-2011

	**Total**	**Place of death**			***p***^**a)**^
		**Home**	**Hospital**	**Nursing home**	
n	58,732	15,597	22,532	20,603	
proportion	1.000	.266	.384	.351	
***Age***					
mean	79.3	73.4	76.5	87.0	***
sd	14.3	17.5	13.2	8.2	
***Sex****(in%)*					
Male	.464	.331	.433	.236	***
Female	.536	.209	.341	.450	
***Deductible class****(in%)*					
Low	.945	.256	.381	.363	***
High (> Swiss francs 500)	.055	.434	.427	.139	
***Managed care plan****(in%)*					
No	.901	.263	.381	.355	***
Yes	.099	.285	.403	.312	
***Private hospital insurance****(in%)*					
No	.809	.264	.363	.374	***
Yes	.191	.274	.473	.253	
***Type of residence****(in%)*					
City	.335	.249	.384	.367	***
Agglomeration	.428	.264	.394	.342	
Rural area	.236	.292	.364	.344	
***Multiple chronic condition****(in%)*					
No	.815	.273	.352	.375	***
Yes (>2 conditions)	.185	.234	.523	.242	
***Latin canton****(in%)*					
No	.748	.275	.367	.358	***
Yes	.252	.238	.434	.329	

Latin cantons include the cantons Fribourg, Geneva, Jura, Neuchatel, Ticino, Vaud and Valais * *P* < 0.10, ** *P* < 0.05, *** *P* < 0.01.

^a) ^Significance level: Kruskal-Wallis-test was used to check for significant differences in age, chi-square-test for differences in the categorical variables.

In terms of the patients’ characteristics and type of additional insurance coverage by place of death, 19.1% of the decedents had supplementary private hospital insurance coverage, 5.5% had a deductible higher than CHF 500 and 9.0% were enrolled in a managed care insurance scheme. 33.5% of the study population lived in a city, 42.8% in an agglomeration and 23.6% in a rural area. 25.2% lived in a Latin canton and 74.8% in a German-speaking canton. Individuals with more than two chronic conditions accounted for 18.5% of the study population.

**Figure 1:**
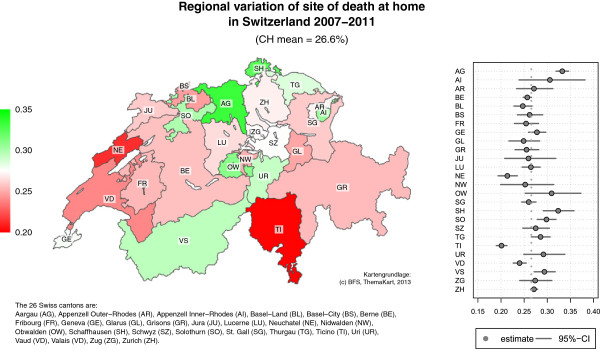
Regional variation of site of death at home in Switzerland, 2007–2011.

With regard to place of death, individuals who died in hospitals accounted for 38.4%, in nursing homes for 35.1%, and those at home for 26.6% of the total. Considerable and significant variation of site of death was observed between cantons (Figure 1). The proportion of people dying at home for example varies between cantons from 22.1% in Ticino TI to 33.3% in Aargau AG (p < 0.001).

We observed a significant difference in age of death with reference to the place of death. People dying in nursing homes were on average 87.0 years old, people dying at home 73.4 years (difference of 13.6 years, p < 0.002).

The difference in age between patient gender remains clear. Men dying at home were 6.3 years younger than women dying at home; in nursing homes the difference amounted to 3.2 years (Table 2). A far greater proportion of women died in nursing homes (45.0%) than men (23.6%), whereas 43% of men died in a hospital and 33.1% at home.

**Table 2:** Proportion of patients according to place of death and gender, 2007-2011

	**Place of death**		
	**Home**	**Hospital**	**Nursing home**
n	15,597	22,532	20,603
proportion	.266	.384	.351
***Males***			
Mean age	70.7	75.2	84.8
n	9,007	11,787	6,436
in%	.153	.201	.110
***Females***			
Mean age	77.07	77.8	88.0
n	6,590	10,745	14,167
in%	.112	.183	.241

The enrollment in a supplementary hospital insurance scheme was associated with hospital as the place of death. 47.3% of the patients with supplementary coverage died in a hospital. With reference to regional differences, we also observed a lower proportion of patients dying at home in Latin cantons. In those cantons the proportion of people dying in a hospital was slightly higher (43.4% vs. 36.7% in German-speaking cantons). Patients with a high deductible were younger on average (p < 0.001), which accounts for the relatively small proportion of 13.9% of patients choosing high deductibles who died in a nursing home.

In general, all observed variables showed a significant association with the place of death. However, the strength of association measured with Cramer’s V illustrates very moderate values (range between 0.00 and 0.22).

### Health care utilization in the last six months of life

We observed mean HCE of 17,686 CHF during the last six months of patients’ lives, with great variability when stratifying for place of death (Table 3). Mean HCE for people dying at home (CHF 11,194) were half as high compared to people dying in hospitals (CHF 23,193). The HCE for people dying in nursing homes lay between these figures (CHF 16,579). Turning to the number of consultations with a general practitioner, we observed a mean of 2.9 consultations, whereas the average number of consultations with a specialist physician was 0.8 in the last six months of life. 55.1% of all patients had at least one consultation with a GP and 26% at least one consultation with a specialist physician. The number of people consulting a GP shows considerable differences in the observed groups. While staying in nursing homes, only every third decedent visited a GP, 68.6% of patients in the hospital group did so.

**Table 3:** Sample characteristics on health care utilization variables, 2007-2011

	**Total**	**Place of death Home**	**Hospital**	**Nursing home**	***p***^**a)**^
Health care expenditures mean (CHF)	17,686.7	11,194.3	23,193.7	16,579.0	***
Standard deviation	15,719.1	13,700.5	19,698.9	8,462.3	
Proportion with values (%)	98.3	93.7	100.0	100.0	
GP consultations mean	2.9	3.0	3.8	1.9	***
Standard deviation	4.5	4.4	4.7	4.2	
Proportion with values (%)	55.1	58.6	68.6	37.8	
Specialist consultations mean	0.8	0.8	1.2	0.3	***
Standard deviation	2.5	2.7	2.9	1.6	
Proportion with values (%)	26.0	27.4	36.3	13.8	
Length of stay hospital mean	16.9	9.7	29.0	9.0	***
Standard deviation	25.6	19.3	30.0	18.5	
Proportion with values (%)	60.8	38.7	99.9	34.7	
Length of stay nursing home mean	58.3	14.2	16.7	137.1	***
Standard deviation	74.8	42.0	45.1	53.0	
Proportion with values (%)	45.6	15.1	17.4	99.5	
Home care costs mean (CHF)	766.9	1,265.7	832.4	317.8	***
Standard deviation	2,369.1	3,357.0	2,221.7	1,294.6	
Proportion with values (%)	28.8	36.1	36.8	14.4	
Different ATC-codes mean	11.3	10.6	13.5	9.5	***
Standard deviation	8.8	9.1	8.8	8.1	
Proportion with values (%)	84.9	84.4	93.8	75.5	

CHF indicates Swiss francs; GP, general practitioner; ATC, Anatomical Therapeutic Classification * *P* < 0.10, ** *P* < 0.05, *** *P* < 0.01.

^a) ^Significance level: Kruskal-Wallis-test was used to check for significant differences between the groups.

A total of 60.8% of all insured had a hospital stay during their last six months of life. The mean duration of stay for all decedents in our sample was 16.9 days. When only considering patients who actually had a stay in hospital, the mean duration was 27.7 days. Here as well, the large difference between the mean lengths of stay (LOS) regarding place of death is primarily caused by people not having a hospital stay. The mean LOS changes from 9.7 (at home) to 25.2, when excluding patients without a hospital stay, which again is in the range of the mean LOS of people dying in hospital (29.0). The mean number of nursing home days was 58.3. Mean home care costs amounted to CHF 766.9. In terms of the number of different drugs consumed (measured by counting distinct ATC codes), the difference between place of death is small, as only around 15.1% of patients did not require any medication. On average, patients were using 11.3 different medications, with a range of 9.5 to 13.5 with reference to place of death (Table 3).

### Place of death

We evaluated the relationship between patient-level characteristics and place of death by means of a multivariate logistic regression model. Table 4 illustrates the OR estimates of the model. The reference of place of death is in each case “home”. The decedent-level variables included in our model account for 17.9% of the variation^e^ of place of death.

**Table 4:** Results of the multinomial logistic regression analysis for decedents in Switzerland regarding “place of death” (reference value: death at home), 2007-2011

	**Coefficient (SE)**	**Odds ratio**^**a)**^	**95% confidence interval**	
hospital : AGE	0.012 (0.001)***	1.012	1.011	1.014
hospital : SEX (female)	0.173 (0.022)***	1.188	1.139	1.240
hospital : PRIV (additional private hospital insurance)	0.102 (0.027) ***	1.107	1.051	1.167
hospital : MC (managed care plan)	-0.095 (0.035)**	0.910	0.850	0.974
hospital : DED (deductible class > Swiss francs 500)	-0.153 (0.042)***	0.858	0.791	0.932
hospital : REG_AGGLO (living in agglomeration)	-0.061 (0.025)*	0.941	0.896	0.988
hospital : REG_RURAL (living in rural area)	-0.217 (0.029)***	0.805	0.761	0.852
hospital : MMORB (> 2 chronic conditions)	0.267 (0.030)***	1.306	1.233	1.385
hospital : log(ATC + 1)	0.318 (0.013)***	1.375	1.339	1.411
hospital : log(GP + 1)	0.097 (0.012)***	1.102	1.076	1.129
hospital : log(SPEC + 1)	0.113 (0.017)***	1.120	1.083	1.158
hospital : log(HOMC + 1)	-0.058 (0.003)***	0.944	0.938	0.950
nursing home : AGE	0.098 (0.001)***	1.103	1.100	1.106
nursing home : SEX (female)	0.626 (0.025)***	1.871	1.780	1.965
nursing home : PRIV (additional private hospital insurance)	-0.253 (0.033)***	0.777	0.728	0.828
nursing home : MC (managed care plan)	-0.084 (0.041)*	0.919	0.848	0.997
nursing home : DED (deductible class > Swiss francs 500)	-0.982 (0.064)***	0.375	0.331	0.424
nursing home : REG_AGGLO (living in agglomeration)	-0.012 (0.029)	0.988	0.934	1.044
nursing home : REG_RURAL (living in rural area)	-0.112 (0.033)***	0.894	0.838	0.954
nursing home : MMORB (> 2 chronic conditions)	0.319 (0.037)***	1.376	1.279	1.480
nursing home : log(ATC + 1)	0.130 (0.014)***	1.139	1.109	1.170
nursing home : log(GP + 1)	-0.340 (0.014)***	0.712	0.692	0.732
nursing home : log(SPEC + 1)	-0.557 (0.025)***	0.573	0.546	0.602
nursing home : log(HOMC + 1)	-0.213 (0.004)***	0.808	0.802	0.815
McFadden adjusted R^2^:	0.17865			
Insample class. error rate	41.38%			
AIC	104937.1			

AGE indicates age at time of death; SEX, sex of patient; PRIV, supplementary private insurance; MC, managed care health plan; DED, deductible class; REG_AGGLO, agglomeration area of residence; REG_RURAL, rural area of residence; MMORB, multiple chronic conditions; ATC, number of different medications; GP, number of consultations with a general practitioner; SPEC, number of consultations with a specialist physician; HOMC, costs of home care.

Standard error (SE) in parentheses.

* P < 0.10, ** P < 0.05, *** P < 0.01.

^a) ^The regression analysis determines the regression coefficient β of each independent variable. In order to obtain the OR of the variables, the coefficients are transformed with exp (β).

Place of death was significantly associated with age, sex, region and multi-morbidity. An elderly woman is more likely to die in a nursing home, whereas a younger man is more prone to die at home. A decedent living in a rural area will presumably die at home and a person in the city in hospital or in nursing home. Multi-morbid patients have a much higher probability of dying in an institution than at home (Hospital death OR 1.306, CI [1.233 to 1.385]; nursing home death OR 1.376, CI [1.279 to 1.480]). Furthermore, the crude numbers of ATC codes are associated with a higher probability of dying either in a hospital or a nursing home. High costs for nursing are associated with a higher probability of dying at home. All of the three insurance variables (private supplementary hospital insurance, managed care health plan and high deductible) are highly significant. Private supplementary hospital insurance coverage shows a higher OR for hospital death, but a decreased probability of dying in a nursing home compared to home. Decedents passing away at home seem to have higher deductibles and are more likely to be enrolled in a managed care health plan compared to decedents who died in an institution. A somewhat contradictory picture is found in terms of number of GP and specialist physician consultations. Both predictors were significantly associated with a higher probability of dying in a hospital. They were also both associated with lower risks of dying in a nursing home.

## Discussion

We found death occurred in Switzerland most frequently in a hospital (38.4% of all cases) followed by nursing homes (35.1%) and dying at home (26.6%), which is generally consistent with other published data [36]. Nearly 26% of men died at home versus 20.9% for women. However, 45% of women died in a nursing home compared to 23.6% of men. We suppose that this fact could mainly be attributed to the higher life expectancy of women. Men can often live longer at home due to the presence of a usually younger partner. They tend to die in hospital after an acute deterioration of their health state. Females spend their last phase of life more frequently without a partner in a nursing home. Additionally, we explored the association of place of death and residential region. People in French- or Italian- speaking cantons appeared to pass away to a greater extent in hospitals (43.4%) than the Swiss average (nursing home 32.9% and home 23.8%). Fischer et al. [36] investigated the place of death in 2001 only in the German speaking part of Switzerland. In comparison with their results, our study found a trend towards dying at home and a shift within institutions from hospitals to nursing homes in the German-speaking part of the country. Similarly, we observed other significant regional differences between age at death among Swiss cantons. This finding is consistent with official data from the SFSO. Further research is needed to determine the extent to which these regional differences are due to patient preferences, supplier-induced demand, differential access to medical services or other factors.

There were substantial and significant differences in the descriptive comparison of health care utilization in the last six months of life between places of death. The mean number of consultations with a general practitioner or specialist as well as the number of different medications and, naturally, the number of days in hospital was consistently highest for the decedents in hospitals. This result can be expected given the generally higher burden of severe illnesses suffered by hospitalized patients. On the other hand, the mean number of consultations with a general practitioner as well as a specialist physician for decedents in nursing homes might not be visible in health insurance data due to aggregated claims data. Therefore, these estimates are possibly biased.

The mean number of different medications consumed by decedents is comparably high in our sample and there is considerable variation, mainly due to people who had no medication at all. Differences in this variable arise when splitting the sample by place of death. In nursing homes we observed an unexpectedly high percentage of people without any medication (24.5%). This number of decedents without any medication might be overestimated as we do not possess any information on medication prescribed in nursing homes.

The majority of people had at least one stay in hospital (60.8%) in the last six months of life and the average length of stay was 16.9 days. However, the differences between the places of death are highly influenced by the number of persons having no stay at all. When restricting the analysis to persons with at least one stay, the mean LOS appeared similar across places of death (27.7 days). The significant duration of hospital stays (25.2 days) for those prior to dying at home was notable. This could point towards the general preference of home as place of death found in prior research [41,42].

Concomitantly and consistent with high expenditures for hospital stays, last six-month HCE are significantly affected by place of death. The mean HCE for hospital deaths, at CHF 23,193.70, is more than twice the mean amount for those dying at home (CHF 11,194.30) and 40% greater than the mean amount for nursing homes (CHF 16,579.0).

Our further analyses are in line with Kelley et al. [32] and show that a substantial portion of the previously described variations in place of death are due to patient-level characteristics. Notably, the proportion of variation explained by our model after patient characteristics were controlled for is however larger than the results presented by Kelley et al.. Place of death is significantly associated with age, sex, region and multi-morbidity. Elderly females have a greater probability of dying in a nursing home, whereas a young male would preferably die at home. Additionally, a decedent living in a rural area will presumably die at home and people in urban areas either in a hospital or nursing home. Persons with multiple chronic conditions have a greater chance of dying in an institution than at home. These findings are in line with earlier studies [14,36,43].

### Strengths and limitations

This study has several strengths. To our knowledge, it is the first empirical investigation, which describes the conditions and health care utilization in the last six months of life with regard to place of death in Switzerland. We use health insurance claims data, which guarantees a uniform data set and presents an ideal basis for the analysis. This study has focused primarily on differences in place of death and health care utilization at the end-of-life, but we cannot comment on the appropriateness of different patterns of care delivered to decedents. In addition, our analysis is based on a period of five consecutive years, which allows us to capture any time effects as well as to reduce standard errors on the interesting variables due to a larger sample.

It is also important to point out to some limitations of our study. From previous unpublished research, we estimate that 2-3% of all claims invoices are paid directly by the patient (e.g. due to high deductibles chosen) and are not reimbursed by the health insurer. This may lead to a possible bias due to a mixture of the different effects in the estimations and missing claims data. Furthermore, we focused on all cases within the mandatory health insurance in Switzerland, which omits people dying from an accident or committing suicide, as these cases might be covered by other insurance policies and the health insurer does not see any referral claims. The claims coming from nursing homes are often set at a flat rate and lack further detailed information. This fact prevented us from observing any medication or medical treatment applied in the nursing home. Therefore, we suppose a slight tendency to underestimate all variables describing health care utilization for people residing in nursing homes. This restriction does not notably affect treatment outside nursing homes (consultations with GPs, outpatient units, pharmacies etc.). To conclude, the place of death is specified by means of the last claim received. The claims contain the date and the duration of the specific treatment, which allows us to compare it with the date of death. The origin of the last claim then defines the place of death. If there are several claims covering the date of death, hospital is taken as place of death. This process makes our data vulnerable to inaccuracies resulting from administrative processes and might lead to a possible overestimation of hospital deaths.

## Conclusion

The importance of nursing homes and patients’ own homes as the place of death will continue to grow in the future. Various international studies confirm this shift, especially towards people’s homes [18,41,42,44]. In order to cope with the growing needs for end-of-life care for ageing populations, the availability of community end-of-life care and non-acute care inpatient facilities must be substantially increased in Switzerland. Knowing the determinants of place of death and patterns of health care utilization of decedents can help decision makers on the allocation of these needed health care services. The study will provide useful data to guide further research and development in this area.

### Endnotes

^a^Classification according to the Swiss Federal Statistical Office (SFSO).

^b^Asthma/chronic obstructive pulmonary disease (COPD), epilepsy, rheumatism, cardiac disorders, Crohn’s disease/ulcerative colitis, gastric disorders, diabetes types I and II, Parkinson’s disease, transplants, cancer, HIV/AIDS and kidney disorders.

^c^WHO Anatomical Therapeutic Chemical (ATC) classification system (WHO Collaborating Centre for Drug Statistics Methodology: *Guidelines for ATC classification and DDD assignment 2011*. Oslo; 2010).

^d^Since dummy variables are dichotomous, a log-transformation makes no sense and is therefore not performed for these independent variables.

^e^According to Hosmer and Lemeshow (2000, p. 167) low R-square values are the norm in logistic regression (Hosmer DW, Lemeshow S: **Assessing the Fit of the model.** In *Applied Logistic Regression.* 2nd edition. New York: Wiley; 2000:167).

## Competing interests

All authors declare that they have no competing interests.

## Authors’ contribution

OR designed the study and drafted the manuscript. AS collected and analyzed the data drawn upon in the study. AB provided input on research methods and edited the manuscript. All authors contributed to the interpretation and prioritization of the findings as well as to writing the paper. All authors reviewed and approved the final manuscript.

## Pre-publication history

The pre-publication history for this paper can be accessed here:

http://www.biomedcentral.com/1472-6963/13/116/prepub
